# Global Longitudinal Strain of the Systemic Ventricle Is Correlated with Plasma Galectin-3 and Predicts Major Cardiovascular Events in Adult Patients with Congenital Heart Disease

**DOI:** 10.3390/medicina56060305

**Published:** 2020-06-22

**Authors:** Alexandra A. Frogoudaki, Ioannis Pantelakis, Vasiliki Bistola, Christos Kroupis, Dionysia Birba, Ignatios Ikonomidis, Dimitrios Alexopoulos, Gerasimos Filippatos, John Parissis

**Affiliations:** 1 Second Cardiology Department, ATTIKON University Hospital, National and Kapodistrian University of Athens, 12461 Athens, Greece; drioannis@yahoo.com (I.P.); vasobistola@yahoo.com (V.B.); dbirba@gmail.com (D.B.); ignoik@gmail.com (I.I.); dalex@med.uoa.gr (D.A.); geros@otenet.gr (G.F.); jparissis@yahoo.com (J.P.); 2 Department of Clinical Biochemistry, ATTIKON University Hospital, National and Kapodistrian University of Athens, 12461 Athens, Greece; ckroupis@med.uoa.gr; 3 Medical School, University of Cyprus, 2029 Nicosia, Cyprus

**Keywords:** systemic ventricular global longitudinal strain, galectin-3, congenital heart disease, arrhythmias

## Abstract

*Backround and Objective*: We sought to assess in adult congenital heart disease (ACHD) patients the prognostic value of plasma galectin-3 (Gal-3) levels and systemic ventricular global longitudinal strain (SV GLS) as well as their association with NTproBNP and arrhythmogenesis. *Materials and Methods*: We studied 58 patients (26 men, mean age 37 ± 16.8 years) with various congenital heart diseases. Patients underwent echocardiogram, 24 h ambulatory ECG monitoring, while NTproBNP and Gal-3 were measured. They were followed up (median of 790.5 days -IQR 350.3 days) and major cardiovascular events (MACE) were recorded. *Results*. Mean Gal-3 levels were 17.07 ± 6.38 ng/m. Plasma Gal-3 was correlated with LogNTproBNP (r = 0.456, *p* = 0.001).Gal-3 levels associated with supraventricular tachycardia (SVT) (*p* < 0.001) and ventricular tachycardia (VT) (*p* < 0.001), but was not associated with MACE (HR 1.018, 95% CI 0.944–1.098, *p* = 0.641).Mean SVGLS in patients with systemic left ventricle was −15.91% ± 4.09%, which was significantly lower compared to patients with systemic right ventricle and patients with single ventricle (−11.42% ± 3.37% and −11.9% ± 5.06%, respectively, *p* = 0.021).SV GLS correlated with plasma Gal-3 (r = 0.313, *p* = 0.027) and logNTproBNP (r = 0.479, *p* < 0.001). SVGLS correlated with VT arrhythmias (*p* = 0.004). NTproBNP predicted MACE (AUC 0.750, *p* = 0.03). SVGLS also predicted MACE (AUC 0.745, *p* = 0.03. In multivariate analysis, SVGLS and logNTproBNP maintained their predictive value (*p* = 0.004 and *p* = 0.009, respectively) *Conclusion*: In ACHD patients, SV GLS was found to predict MACE independently from NTproBNP and correlated with VT. Gal-3 correlated with NTproBNP and SVGLS as well as SVT and VT, but has not been shown to bear significant prognostic potential.

## 1. Introduction

Heart failure (HF) and arrhythmias are the most frequent complications throughout the life of adult congenital heart disease (ACHD) patients. They often coexist and represent the commonest causes of morbidity and mortality in this population. While after surgical interventions patients often experience relatively uneventful childhood courses, arrhythmias emerge in adulthood with a burden which increases significantly with age [1,2,3]. The cornerstone of the wound-healing process is scar formation, mediated by activated fibroblasts (myofibroblasts) that synthesize excess collagen leading to myocardial fibrosis. Myocardial fibrosis contributes to the progression of heart failure and is linked to poor outcome in patients with cardiovascular disease. Consequently, biochemical indices of myocardial fibrosis have been suggested as potential markers of disease progression and therapeutic response. Plasma galectin- 3 (Gal-3), a member of the carbohydrate-binding protein family of lectins, is currently being studied as a potential novel biomarker for cardiac fibrosis and adverse remodeling in heart failure. Experimental studies have shown that Gal-3 is synthesized by activated macrophages and mediates profibrotic processes in rodent models of heart failure [4]. In echocardiography, the term ‘strain’ is used to describe local shortening, thickening and lengthening of the myocardium as a measure of regional left ventricular (LV) function. Even in early stages of cardiac disease, speckle tracking echocardiography (strain imaging) can be of considerable value in the diagnostic evaluation and in defining prognosis. Global longitudinal strain (GLS) has been shown to predict cardiovascular outcomes possibly superior than LV ejection fraction (LVEF) in a number of cardiac disorders [5]. Three dimensional (3D) GLS has been moderately associated with the extent of myocardial fibrosis in magnetic resonance imaging (MRI), with a promising potential role in ruling out prognostically relevant fibrosis as detected by LGE [6]. Moreover, moderately and severely impaired LV GLS has been associated with poor outcomes in heart failure patients with QRS width <130 ms [7]. Impaired LVGLS has also been associated with increased collagen synthesis markers indicative of fibrosis in hypertensive patients [8,9].

Therefore—in ACHD patients without worsening symptoms of heart failure or recent hospitalizations—we aimed to assess the following: (1) The prognostic value of plasma Gal-3 levels and systemic ventricle (SV) GLS, (2) The association of plasma Gal-3 levels with GLS of the systemic ventricle, (3) The association of plasma Gal-3 levels and GLS with supraventricular and ventricular arrhythmias, (4) The association of plasma Gal-3 levels with NTproBNP, an established prognostic marker in ACHD patients.

## 2. Methods

The study was prospective and conducted at the Adult Congenital Heart Disease Clinic, Second Cardiology Department, ATTIKON University Hospital, Athens, Greece. The study protocol was approved by the Institutional Review Board (2/2-2-2012). In addition, all participants gave their written informed consent. One-hundred-nine patients seen at the outpatient clinic from December 2014 until February 2016, were screened for inclusion in the study. Fifty eight, patients with CHD (26 men, mean age 37 ± 16.8 years), without symptoms of heart failure deterioration or recent hospitalizations (during the previous 3 months), able to understand and sign the informed consent and available to undergo all tests, with normal or at most mildly impaired renal function (GFR ≥ 60 mL/min) were included. Physical examination and a standard 12-lead electrocardiogram were performed in all patients. NYHA class was recorded.

All patients underwent 2D echocardiogram and 24 h ambulatory ECG monitoring. Peripheral venous blood samples were drawn in order to measure plasma GAL-3 and serum NT-proBNP levels. All tests were conducted within a time interval of 7 days. Echocardiography studies were performed with Vivid 7 (GE Medical Systems, Horten, Norway) ultrasound system. All studies were digitally stored in a computerized station (Echopac 202 GE, Horten, Norway) and analyzed by two observers, blinded to clinical and laboratory data. Three patients had inadequate images for analysis and thus were excluded from the study.

Measurements were performed according to the current guidelines of American Society of Echocardiography and the European Association of Cardiovascular Imaging [10]. Interventricular septum, LV posterior wall thickness, LV end-diastolic diameter, LV end-systolic diameter and systemic atrial diameter were measured. End-diastolic and end-systolic areas were measured from the apical four-chamber and two-chamber views for the calculation of LV ejection fraction (EF) using the modified biplane Simpson’s method. Simpson’s method was also used to measure systemic right ventricular (RV) EF. Markers of RV function included tricuspid annulus plane systolic excursion (TAPSE) measured with M-Mode of the tricuspid annulus from focused four-chamber view and TDI-derived peak systolic velocity of the tricuspid annulus.

Subpulmonary ventricle systolic pressure was measured when possible, by using continuous wave Doppler to obtain the peak subpulmonary atrioventricular valve regurgitant velocity, combined with right atrial pressure, as estimated by size and respiratory variation of inferior vena cava.

By means of a dedicated software package (Echopac, 202 GE Medical systems, Horten, Norway), LV myocardial deformation was calculated using manual tracing speckle tracking analysis (Q analysis), according to current recommendations [11]. Frame rates of >50 frames per second were used, after optimal sector width and depth adjustments. We calculated LV global peak longitudinal systolic strain (LVGLS) as means of the 17 ventricular segments from the four-chamber, two-chamber and three-chamber apical views [11]. Right ventricular peak longitudinal systolic strain (RVGLS) was calculated as means of the six segments (three of the RV free wall and three of the intraventricular septum) from the apical four chambers view. The inter- and intra-observer variability of LVGLS was 8% and 10%, respectively.

Laboratory measurements: Patient blood samples were drawn in BD Vacutainer gel clot tubes and were centrifuged within 30 min. The recovered sera were stored at −20 °C until the analysis day.

ΝΤ-proBNP was measured with the standardized Roche electrochemiluminescent method in a Cobas e601 platform.

We measured Gal-3 with a commercial two-step chemiluminescent immunoassay (Architect Galectin-3, Abbott Laboratories, Chicago, IL, USA) in the automated i2000SR Architect platform. The assay employs two monoclonal antibodies for the detection of the molecule (M3/38 and 87B5) and is designed to have an imprecision of ≤10% total CV within its measurement range 4.0–114.0 ng/mL. Hemolyzed samples were excluded and patient measurements were performed in duplicate along with appropriate control samples.

Patients were followed by clinical visit or phone call and major cardiovascular events (MACE) were recorded. Death, hospitalization due to cardiac causes, worsening functional class or cardiac interventions were defined as MACE.

Statistical analysis was performed with SPSS v.21 (IBM Corp.). Baseline characteristics of the study population are described using frequencies with percentages for categorical variables and means ± SD for normally distributed continuous variables and median (interquartile range, IQR) for non-normally distributed variables. Comparisons of numeric variables among SVT and VT subgroups were performed using one-way ANOVA. Correlations between variables were tested with Pearson test. Prognostic potential of SV GLS and biochemical markers Gal-3 and NTproBNP were tested by univariable Cox regression analysis. Markers shown to have significant prognostic association in univariate analysis were entered into multivariable Cox regression analysis adjusting also for age. Log transformed NTproBNP was entered in regression analysis. Receiver-operating curve analysis was used to define optimal cutoff levels and respective sensitivity and specificity of SV GLS and NTproBNP to predict MACE. Statistical significance was considered at *p* value <0.05.

## 3. Results

A total of 58 patients with ACHD without deterioration of heart failure symptoms and without recent hospitalizations (previous 3 months), were included (26 men, mean age 37 ± 16.8 years). Forty-nine patients had systemic LV, 5 had systemic RV and 4 had single ventricle (SiV) physiology. All SiV patients had LV morphology. Diagnosis and demographic–clinical data of the patient population are described in Table 1 and Table 2, respectively.

### 3.1. Arrhythmias

Patients were divided in 3 groups according to the occurrence of supraventricular tachycardia (SVT) in 24 h ambulatory ECG monitoring: Group A: no SVT (*n* = 16, 27.5%), Group B: 1–2 episodes of SVT or SV extra systoles (*n* = 27, 46.5%), Group C: multiple SVT episodes or atrial fibrillation (*n* = 15, 25.8%); and in 3 groups according to the occurrence of ventricular tachycardia (VT): Group A’: no VT, (*n* = 15, 25.8%), Group B’: extrasystoles or couplets (*n* = 32, 55.1%), Group C’: triplets or non-sustained VT or more than 1000 extra systoles(*n* = 11, 18.9%).

### 3.2. Biomarkers

Mean Gal-3 levels were 17.07 ± 6.38 ng/mL. Median NTproBNP was 119 pg/mL (IQR 174.3 pg/mL). Median NT-proBNP was 87.5 pg/mL (range 46.9–114 pg/mL, *p* = 0.02) in a sex and age matched healthy control group.

Plasma Gal-3 was positively correlated with LogNTproBNP (r = 0.456, *p* = 0.001) and systemic atrial diameter (r = 0.316 *p* = 0.025).

Gal-3 levels differed significantly between patient subgroups defined by the presence and severity of SVT (group A: 12.621 ± 4.148 ng/mL, group B: 17.092 ± 5.960 ng/mL, group C: 22.427 ± 5.95 ng/mL, *p* < 0.001) and VT (group A’: 12.631 ± 4.014 ng/mL, group B’: 16.750 ± 5.275 ng/mL, group C’: 23.456 ± 7.410 ng/mL *p* < 0.001).

Gal-3 was not correlated with age, SV ejection fraction and sub-pulmonary ventricular pressure.

### 3.3. Global Longitudinal Strain

Mean systemic ventricle GLS (SVGLS) in patients with systemic LV was −15.91% ± 4.09%, which was significantly lower (closer to normal values) than SVGLS in patients with systemic RV and patients with SiV (−11.42% ± 3.37% and −11.9% ± 5.06%, respectively, *p* = 0.021).

In 30 healthy controls matched for age and sex LVGLS was measured −22.1 ± 1.9, *p* = 0.03, while RVGLS was measured −23.9 ± 8.9, *p* < 0.001 in our institution’s echo laboratory.

SV GLS (LV, RV and SiV) correlated positively with plasma galectin-3 (r = 0.313, *p* = 0.027), logNTproBNP (r = 0.479, *p* < 0.001) and systemic atrial diameter (r = 0.392, *p* = 0.02).

SVGLS did not differ significantly between patient subgroups defined according to SVT status, but was significantly impaired in subgroups of increasing severity of VT arrhythmias (group A’: −18.36% ± 2.71%, group B’: −14.22% ± 4.46%, group C’: −13.72% ± 3.72%, *p* = 0.004).

### 3.4. Clinical Outcomes and Prognostic Associations

Patients were followed for a median of 790.5 days (IQR 350.3 days). Reported follow-up was up to October 2018 for patients without any MACE, but was performed until an event occurred in patients with MACE. Twenty patients had a MACE (1 cardiovascular death, 7 cardiac interventions, 8 hospitalizations due to cardiac reasons, 4 worsening of functional class).

Cardiac interventions included aortic valve replacement, peripheral pulmonary arteries angioplasty, atrial septal defect closure, coronary artery bypass grafting combined with pulmonary valve replacement, transcatheter pulmonary valve replacement with Melody valve, supravalvular pulmonary angioplasty and pacemaker insertion.

Four patients were hospitalized due to heart failure, 3 patients due to arrhythmias and one because of pulmonary embolism.

Gal-3 was not associated with MACE in univariable analysis (HR 1.018, 95% CI 0.944–1.098, *p* = 0.641).

**Figure 1:**
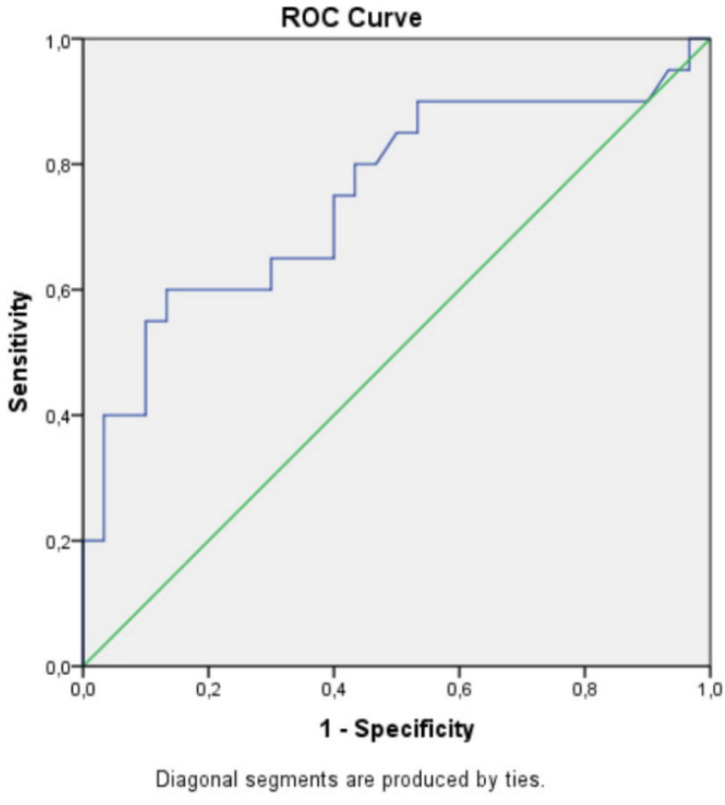
A cutoff value for NTproBNP of 112 pg/mL predicted major cardiovascular events (MACE) with 75% sensitivity and 60% specificity (AUC 0.750, *p* = 0.03).

**Figure 2:**
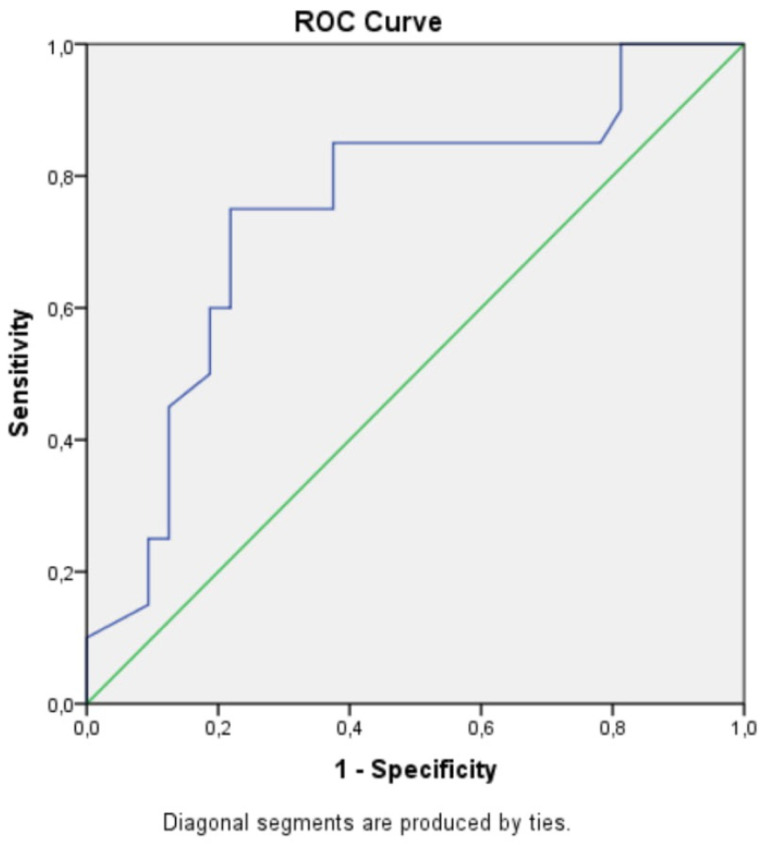
A cutoff value of mean systemic ventricle GLS (SVGLS)−14.45% predicted MACE with 72% sensitivity and 78% specificity (AUC 0.745, *p* = 0.03).

SVGLS and logNTproBNP were both associated with MACE in univariate Cox regression analysis (HR 1.23, 95% CI 1.099–1.378, *p* = 0.0003; and HR 5.313, 95% CI 2.204–12.806, *p* = 0.0002, respectively). A cutoff value for NTproBNP of 112 pg/mL predicted MACE with 75% sensitivity and 60% specificity (AUC 0.750, *p* = 0.03), (Figure 1). For SVGLS, a cutoff value of −14.45% predicted MACE with 72% sensitivity and 78% specificity (AUC 0.745, *p* = 0.03), (Figure 2).

In multivariate analysis, SVGLS and logNTproBNP maintained their predictive value when both were entered in the model and adjusted for age (HR 1.208, 95% CI 1.06–1.376, *p* = 0.004; and HR 4.058, 95% CI 1.429–11.521, *p* = 0.009, respectively).

## 4. Discussion

In the present study, we have shown that SVGLS predicted MACE independently from NTproBNP in adult patients with CHD followed for at least one year.

In our cohort, Gal-3 was positively correlated with systemic atrial diameter, NTproBNP, SVT and VT indicating, as a marker of fibrosis, that systemic ventricular and atrial fibrosis may contribute to increased SV end-diastolic pressure in ACHD patients, although it did not predict MACE in this population.

In a recently published study [12] that included 591 ACHD patients, Gal-3 was positively associated with age, use of cardiac medications, higher NYHA class, loss of sinus rhythm, ventricular dysfunction (both systolic and diastolic) and NT-proBNP. Gal-3 was significantly associated with adverse cardiovascular events in univariable analysis. However, this association was negated in multivariable analysis after adjustment for NT-proBNP.

In another study that included 70 patients with SiV post Fontan type operation, GAL-3 levels were increased compared to age- and sex-matched controls and associated with increased risk of non-elective cardiovascular hospitalization or death [10].

In the present study, SV GLS was significantly impaired in systemic RV and SiV compared to systemic LV patients. In a recent publication, normal values of RV strain were impaired compared to our cohort (−21.5% ± 3.2% vs. −11.42% ± 3.37%) [13]. Similarly, LV GLS strain values in our study are impaired according to EACVI-proposed normal values [14].

LV GLS has been proven of prognostic value in several types of heart disease and thus its use has been implemented in clinical practice [15]. Reduced LV GLS possibly reflects increased LV fibrosis as GLS has been shown to be an index of LV global function that is strongly correlated with the extent of myocardial fibrosis in patients with advanced systolic HF requiring heart transplantation [16] and in hypertensive heart disease [8]. Moreover, myocardial deformation parameters derived from standard MRI protocols has been correlated with symptoms and clinical deterioration in patients with ToF and also predicted adverse outcomes [17]. In another cohort of patients with systemic RV, fibrosis as assessed by MRI was associated with worse clinical outcome and atrial arrhythmias [18].

Echocardiography-derived deformation [19,20,21] has been utilized for the assessment of systemic and/or subpulmonary ventricle in patients with ToF and atrial septal defect.

Significance of systemic RV GLS as a prognostic marker in CHD has been addressed previously. In adults with atrial switch TGA, RV GLS was significantly reduced compared to controls was correlated to subpulmonary ventricular function, while it also predicted adverse clinical outcomes [22]. In a study, RV GLS was associated with elevated NT-proBNP and tended to correlate with worsening NYHA class [23]. In a recent MRI study global circumferential strain was significantly different for those with systemic RV EF above and below 45%, while GLS was not [24].

In our cohort Gal-3 a biomarker reflecting fibrosis and SV GLS an imaging derived parameter with strong prognostic value, that also reflects fibrosis correlated. SV GLS was also correlated with NTproBNP, systemic atrial diameter (which reflects increased systemic ventricle end diastolic pressure) and ventricular tachycardia, suggesting that fibrosis of the systemic ventricle contributes to ventricular arrhythmogenesis in ACHD. Furthermore, we have shown that SV GLS predicted MACE independently from NTproBNP.

On the other hand, Gal-3 may reflect fibrosis in ACHD patients, but its predictive value is low compared to NTpro BNP and SVGLS, a finding that is in accordance with literature.

The present study has shown for the first time the association of Gal-3 with SVGLS, SVT and VT arrhythmias in ACHD.

It has been previously shown that LVGLS may predict life threatening arrhythmias (VT) and death in a cohort of tetralogy of Fallot (ToF) patients [25].

As arrhythmia is an important determinant of prognosis in ACHD and fibrosis presents one of the underlying mechanisms of arrhythmia, early detection of fibrosis with either biomarkers or imaging may assist in therapeutic management, and therefore favorably impact prognosis of this relatively young and growing population.

### Limitations

This study has included a small number of patients, who, however, consisted of consecutive outpatients without deterioration of heart failure symptoms and without recent hospitalizations, with normal or at most mildly impaired renal function followed at the ACHD clinic of our institution. Heterogeneity of ACHD patients is another limitation. A broad spectrum of CHD was included in our study ranging from simple atrial septal defects to complex cyanotic congenital heart disease. Nevertheless, this population is highly representative of an ACHD outpatient population followed at tertiary ACHD clinic.

Furthermore, despite the vast spectrum of underlying cardiac anatomy, many types of ACHD share common features with respect to pathophysiology, symptoms and natural history.

Finally, we didn’t perform serial 24 h ambulatory ECG monitoring tests in all patients as our purpose was to assess the arrhythmic burden at the point 0, when strain, NTproBNP and galectin-3 calculations were performed, and explore the possible associations. Only patients with clinically relevant arrhythmia had repeat 24 h ambulatory ECG monitoring.

## 5. Conclusions

In ACHD patients, systemic ventricle global longitudinal strain was found to predict MACE independently from NTproBNP and correlated with ventricular arrhythmias. Although Gal-3 was correlated with NTproBNP and SVGLS as well as VT and SVT arrhythmias, it has not been shown to bear significant prognostic potential.

## Figures and Tables

**Table 1:** Congenital diagnoses of patient population.

Main CHD Diagnosis	*N* (%)
Atrial Septal Defect	19 (32.7)
Ventricular septal defect	3 (5.2)
Aortic stenosis/regurgitation/LVOT abnormality	3 (5.2)
Coarctation of the aorta	4 (6.9)
Pulmonary stenosis	3 (5.2)
Pulmonary regurgitation	1 (1.7)
Tetralogy of Fallot	14 (24.1)
Transposition of the great arteries	2 (3.4)
CC Transposition of the great arteries	3 (5.2)
Single ventricle	4 (6.9)
Eisenmenger syndrome	2 (3.4)

**Table 2:** Demographic and clinical characteristics of patient population.

Sex (male), *n* (%)	26 (44.8)
Age (years)	37 ± 16.8
NYHA class, *n* (%)	
I	29 (50)
II	26 (44.8)
III	3 (5.2)
Sinus rhythm, *n* (%)	52 (89.6)
Systemic ventricle, *n* (%)	
LV, *n* (%)	49 (84.4)
RV, *n* (%)	5 (8.6)
SiV, *n* (%)	4 (6.9)
SVEF, %	55 ± 6
Subpulmonary ventricular pressure, mmHg	46 ± 22
SVGLS, %	−15,247 ± 4.32
Plasma galectin, ng/mL	17.07 ± 6.38
NTproBNP, pg/mL (IQR)	119 (174.3)
